# Characterization of Industry 4.0 Lean Management Problem-Solving Behavioral Patterns Using EEG Sensors and Deep Learning

**DOI:** 10.3390/s19132841

**Published:** 2019-06-26

**Authors:** Javier Villalba-Diez, Xiaochen Zheng, Daniel Schmidt, Martin Molina

**Affiliations:** 1 Fakultät Management und Vertrieb, Hochschule Heilbronn Campus Schwäbisch Hall, 74523 Schwäbisch Hall, Germany; 2 Departament of Artificial Intelligence, Escuela Técnica Superior de Ingenieros Informáticos, Universidad Politécnica de Madrid, 28660 Madrid, Spain; 3 Departament of Business Intelligence, Escuela Técnica Superior de Ingenieros Industriales, Universidad Politécnica de Madrid, 2006 Madrid, Spain; 4 Saueressig GmbH + Co. KG, Gutenbergstr. 1-3, 48691 Vreden, Germany

**Keywords:** EEG sensors, manufacturing systems, problem-solving, deep learning

## Abstract

Industry 4.0 leaders solve problems all of the time. Successful problem-solving behavioral pattern choice determines organizational and personal success, therefore a proper understanding of the problem-solving-related neurological dynamics is sure to help increase business performance. The purpose of this paper is two-fold: first, to discover relevant neurological characteristics of problem-solving behavioral patterns, and second, to conduct a characterization of two problem-solving behavioral patterns with the aid of deep-learning architectures. This is done by combining electroencephalographic non-invasive sensors that capture process owners’ brain activity signals and a deep-learning soft sensor that performs an accurate characterization of such signals with an accuracy rate of over 99% in the presented case-study dataset. As a result, the deep-learning characterization of lean management (LM) problem-solving behavioral patterns is expected to help Industry 4.0 leaders in their choice of adequate manufacturing systems and their related problem-solving methods in their future pursuit of strategic organizational goals.

## 1. Introduction

In the search of operational excellence in an Industry 4.0 context, manufacturing leaders constantly face a myriad of ever-changing challenges. They make thousands of choices, often under pressure, between alternatives with different overall value outcomes, and thereby exercise their ability to make adequate decisions. This ultimately determines their individual and organizational success. Operational excellence is a business discipline whose original main driver is the continuous improvement of processes [1] while encompassing other disciplines such as lean management (LM) [2], its combination with six sigma [3], scientific management [4], and organizational design [5]. Specifically, LM is a management discipline that supports the operational excellence effort by focusing on maximizing the value [2] of complex networked value streams [4] by systematically reducing internal process variability [6,7]. The LM system is based on different variations of the Shewart–Deming problem-solving quality control loop [8]. LM thus enables organizational leaders to attain operational excellence and cope with the socio-technical challenges that environmental complexity poses by applying a set of problem-solving behavioral patterns to several challenges—such as just-in-time production, total quality management [9], or service quality level increase [10], for instance. For these reasons, LM was chosen as a preferable framework to try to better understand problem-solving behavioral patterns of individuals within complex manufacturing contexts.

Although many LM problem-solving behavioral patterns are reported to have been implemented with value-stream performance increase [11,12,13,14,15], there is still much controversy as to which discriminating characteristics make some of these problem-solving behavioral patterns better than others and why [16]. The reason for this might be that scholars have not provided quantifiable evidence yet of the process owners‘ real brain activity when performing such tasks. This could help to provide an understanding of the similarities and differences between the different proposed behavioral patterns. In the absence of such an analysis, the discussion remains subject at best to inference and at worst to trends or opinion. Such awareness is of utmost importance to facilitate the decision of which behavioral patterns should potentially be used during the implementation of different manufacturing systems.

This work aims to use modern sensor technology located on the human brain to capture signals that help characterize the cortical activity of individuals performing problem-solving tasks in Industry 4.0 environments. The technology based on non-invasive low-cost sensors that offer neuroimaging in real environments such as industrial ones is not sufficiently developed [17,18]. The sensors used in real environments must guarantee the necessary comfort, low invasiveness, and high reliability. For this reason, not all devices available on the market are suitable for these applications [19]. On the other hand, the combination of this hardware with soft sensors based on artificial intelligence that allow increase of the low signal-to-noise ratio (SNR), is a promising line of research when combining brain–computer interface algorithms with biosignal acquisition technologies [20]. This represents undoubtedly a strength of the work presented. The overarching goal of this research is to offer Industry 4.0 leaders a better understanding of the brain processes underlying problem-solving behavioral patterns, as well as to highlight possible management implications when choosing the most appropriate manufacturing systems to achieve their strategic objectives.

**Figure 1:**
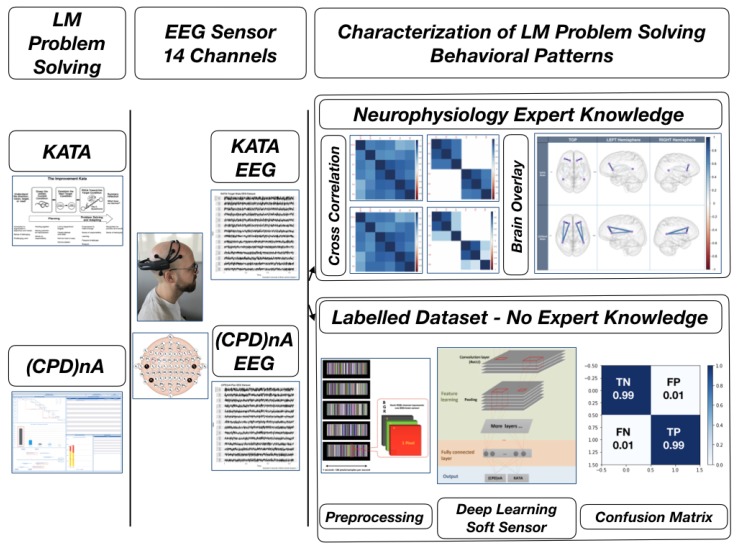
Graphical Abstract.

As the graphical abstract shows in Figure 1, this is achieved by means of a case study within an Industry 4.0 automotive Japanese manufacturing facility in which several LM process owners are asked to solve value-stream-related problems with two specific LM behavioral patterns while being subject to non-invasive low-cost sensors electroencephalographic (EEG) measures. Subsequently, two methods are used to perform a characterization of the tasks. One is based on expert neurophysiological hypotheses. Other is based on a deep-learning (DL) soft sensor that performs the classification of pre-processed labelled EEG signals with a 99% accuracy rate.

After placing the study in a broad context, exploring the importance of the problem, outlining the purpose and its significance, as well as highlighting the relevance of the achieved results, the structure of the paper hereinafter is structured in order to ensure clarity in the presentation, replication of the results obtained, and a proper framing in the ongoing global research context. First, Section 2 starts by providing a brief framework through key publications on neurological goal-directed decision-making, on LM methodologies and outlining the research hypotheses. Second, the Materials and Methods Section 3 describes in detail how field research was conducted with aid of a case study. Additionally, the dataset of the case study, and notebook code is made available on an Open Access Repository to allow for verification and ensure replicability. Third, the Results and Discussion Section 4 summarizes and discusses the results obtained. Finally, Section 5 outlines several managerial conclusions, and future expected management implications from a broad operational excellence endeavor perspective.

## 2. Literature Review

Industry 4.0 leaders make decisions in an ever-changing environment and in order to choose between value outcomes of their LM oriented actions they need to be aware that optimal decision-making requires three main characteristics [21]: self-control, active working memory, and adaptive modulation of this value signal. In neurological terms, such functions are understood as ***executive goal-directed decision-making*** and are neurologically managed by the prefrontal cortex (PFC). Neuroscientists such as Miyake [22] have shown that some skills crucial for Industry 4.0 decision-making constitute the PFC function:

1. Inhibition
  A capacity to resist to distraction. While solving problems in industrial shopfloors with high levels of potential distractions [23], it is important to focus on the most important root-causes of value-stream variability and discard less relevant information.
2. Shift
  A capacity to shift smoothly from one task, routine, or context to another. When dealing with highly interdependent complex processes, typical for instance of re-configurable manufacturing systems [24], it is important to shift within several levels of complexity to flexibly conduct an analysis in a multidimensional complex environment.
3. Working Memory
  A capacity to hold and manipulate multiple ideas. Within a manufacturing environment with multiple interconnected processes, it is necessary to accurately hold a significant amount of relevant information when realizing problem-solving tasks [25].

The PFC is a complex region of the brain that allows us to adapt to an ever-changing environment [26]. It seems, therefore, more than plausible to hypothesize that the brains of those industrial process owners who practice LM should present a high level of PFC activity. However, do they? The fact of the matter is that this is not really known with any certainty. Therefore, it is imperative to explore the unique PFC features in the LM context to better understand LM problem-solving behavioral patterns. This is the main motivation of this paper. To date there have been several systematical studies on the relationship of brain activity to high-level complex cognitive tasks [27,28] and our aim is to deepen this body of knowledge.

Decades of neuropsychological research has related the PFC of the brain processes that guide goal-directed and purposeful behavior: goal-directed spatial navigation [29,30], goal-directed food choice in obesity [31], cognitive rehabilitation [32], reward-based learning [33,34], decision-making impairment [35], response inhibition to stimuli [36,37], etc. PFC guided top-down modulation underlies our capacity to attend to significant and discard other less relevant stimuli [38]. This implies a hierarchical guidance of our thoughts, actions, and emotions. There are several organizational principles to distinguish between functions of the lateral and medial areas of the PFC:

1. Outer/Inner World Representation
  One of the first of these approaches in primates considers that the lateral PFC area represents the outer world related cognition and that the medial and ventral PFC represent our inner emotional world [39].
2. Abstract/Social Cognition
  Another approach considers that the PFC has an anterior-posterior organization in which anterior areas are involved in abstract information processing [40], such as metacognition, whereas more posterior regions are involved in social cognition [41].
3. Inhibition/Generative
  Aron [42] suggest that the PFC presents a hemispheric lateralization in which the right hemisphere inhibits improper emotions or actions, whereas the left hemisphere concentrates on generative processes. These results are in the same line of those exposed in the avoidance (BIS) vs. approach (BAS) resting state and personality component theory [43]. They explain how high levels of BAS explain high levels of cortical activity in the right hemisphere while in the resting state and in experimental conditions with positive stimuli. In contrast, high BIS levels indicate cortical activity in the right hemisphere while in the resting state and under experimental conditions with negative stimuli.
4. Context-Dependent Goal Modulation
  More recently, researchers have recognized that context-dependent, goal-directed behavioral control and decision-making ‘involves constant reciprocal and dynamic communication between PFC cortices and posterior brain regions’ [44]. Specifically, the ventromedial PFC supplies the basis for goal-directed decision-making [45] and the context-dependent functionality originates in a modulation of the ventromedial PFC by the dorsolateral PFC [46]. Correlative interaction between such brain regions, ‘enable goal modulation of brain activity based on goal states’ [47,48].

Subsequently, malfunctioning connectivity between the ventromedial PFC and dorsolateral PFC regions has been associated with ‘poor context-dependent, goal-directed modulation and distorted problem-solving behavioral patterns’ when aging [49]. Two relevant examples for organizational leaders and decision makers in the context of operational excellence of this can be found in transient (stress) or permanent (psychopathy) neural conditions:

1. Stress
  Manufacturing leaders are constantly under environmental pressure. It has been proven by that ‘exposure to uncontrollable stress, acute, or chronic, causes temporal loss of PFC cognitive functions’ [50], which leads to poor decision-making. The fact that even mildly ’acute uncontrollable stress induces a rapid and dramatic loss of PFC cognitive abilities’ is particularly relevant for organizational leaders and decision-making when dealing with subordinates [51].
2. Psychopathy
  Psychopaths present a demonstrated, reduced neural synchronization between ventromedial PFC and dorsolateral PFC while engaged in cognitive tasks with an emotional component [52]. Such cerebral functional configuration seems to (1) suppress decentralized information that is a-priori irrelevant to the goal at hand [53] and (2) lead to a predisposition of moral judgement impairment [54]. This might be why there is a disturbingly high number of individuals with such a personality trait who assume leadership roles [55].

However, shifting attention between different perspectives or behavioral flexibility, depending on the context, is the key aspect to consider here. According to [56], behavioral flexibility is subserved by the dorsolateral PFC, but these scholars demonstrate that the temporoparietal junction (TPJ) plays a coordinated role with dorsolateral PFC in stimulus-driven attention shifting. A combined activity of dorsolateral PFC and TPJ predicts flexible context-dependent cognitive shifting. As such, changes in the reciprocal coordinated functional connectivity of these brain regions may provide a powerful marker with which to assess brains‘ ability to perform flexible context-dependent mapping from sensory evidence. Furthermore, the cost and limitations, depending on the task complexity of the ventromedial PFC-dorsolateral PFC [57] of goal-directed modulation and dorsolateral PFC-TPJ [56] context-dependent cognitive flexibility support the hypothesis that its engagement is only activated if the behavioral task at hand requires it.

The fundamental behavioral task of LM is the systematic implementation of the Shewart–Deming cycle or PDCA (Plan-Do-Check-Act) [2,4]. However, there are numerous interpretations of such a core common denominator, as shown in [14]. In behavioral terms, all of them are, by definition, goal-directed. They can be qualitatively categorized in one of two main classes, depending on their context-dependent valuation:

1. Context-Independent
  These typically present a fixed target-state condition or future-state that is to be achieved by the subject. Some examples are [11,15,58,59].
2. Context-Dependent
  This provides a direction (HOSHIN) of improvement, but does not set any specific goal. Examples include some Japanese interpretations of PDCA [14,23,60,61].

**Figure 2:**
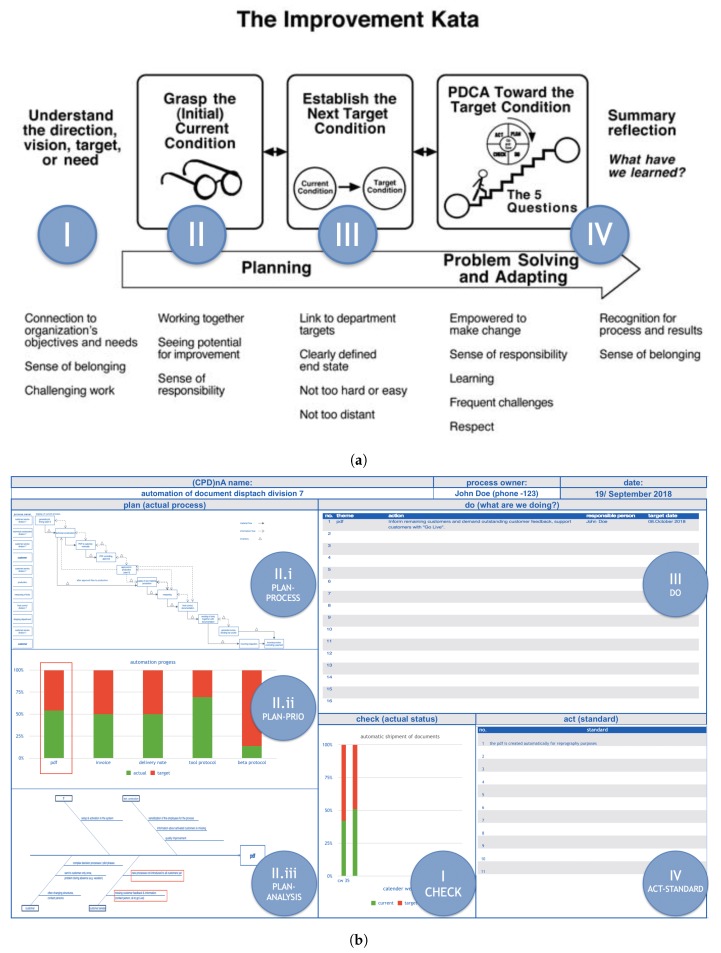
LM Goal-Directed problem-solving behavioral patterns [16]. (**a**) KATA [11]. (**b**) (CPD)nA [14] by Norbert Rosenfeld. Saueressig GmbH. Vreden. Germany. Reproduced with permission.

Within this frame, the scope can be narrowed now by seeking to determine which neural processes present a high correlation, while performing two specific LM problem-solving behavioral patterns, *KATA* [11] and *(CPD)nA (Check-Plan-Do-...n-times-...-Act)* [14], as shown in Figure 2:

*KATA* [11] is a standardized behavioral pattern that can be summarized in four steps:
  I Set direction. Decide in which direction there can be improvement.
  II Understand the current state. Create a common understanding of the factual reality of the value stream at hand.
  III Establish target condition. Fix a target state for the subject to achieve.
  IV Perform PDCA towards the target condition. Systematically and iteratively approach the target state.
  *KATA* fixes the subject’s attention to a certain set of target-state conditions (Step 3) and does not permit these to be changed until they are achieved (through Step 4). This does not permit the subject to shift between contexts, for his/her mind is concentrated on target-state achievement.
*(CPD)nA* [14] derives from Japanese interpretations of continuous improvement [61] and its standardized behavioral pattern can be summarized in four steps:
  I Check. Decide how to measure success.
  II Plan.
    - Plan-Process. Separate what is known from what is unknown in the value stream.
    - Plan-Priority. Understand the main sources of value-stream variability.
    - Plan-Root Cause Analysis. Analyze the main source of internal process variability in search of its root cause.
  III Do. Define an action to eliminate the source of internal process variability.
  IV Act. Standardization of the best-known way to carry out the process.
  *(CPD)nA* does not set any specific target state, and instead, encourages continuous improvement of the given success measurement (calculated from the Check) based solely on the knowledge of the current state of the value stream. This permits the subject to shift flexibly between contexts to adapt his/her behavior to the current state condition.

**Figure 3:**
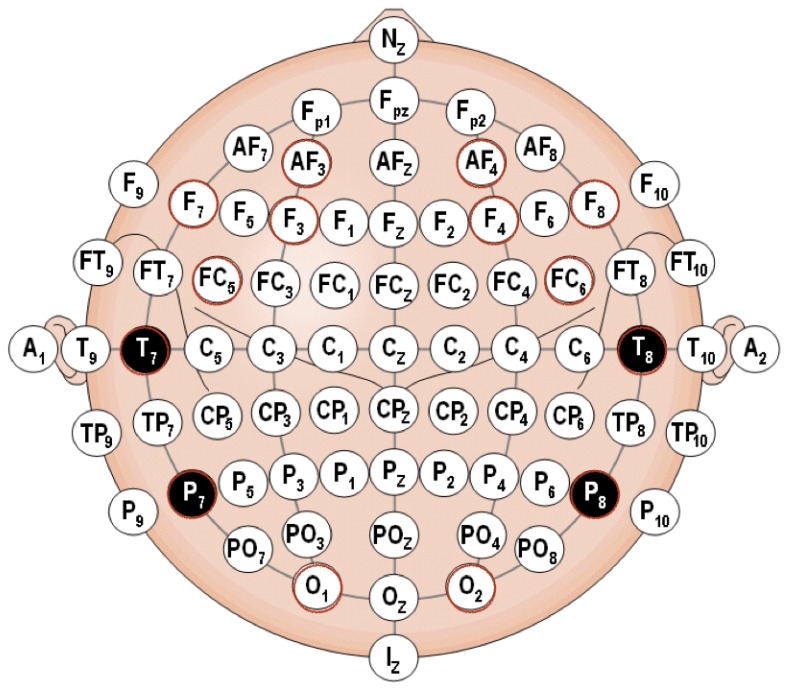
AES EEG electrode standardized nomenclature.

The use of non-invasive brain EEG signals through wearable technology has been previously proven helpful [62]. Some of the multiple applications of this technology can be found in task recognition [17], evaluation of driver vigilance [63,64,65], characterization of focused attention and working memory [66], emotional states [67] and stress [68,69] assessment, stimulus recognition [70], cognitive workload classification [71] or user’s states assessment [72], and for the formulation of control commands [73]. Several electroencephalographic (EEG) standards exist in the sensor characterization on the human brain and in this paper the American Electroencephalography Society [74] standard was chosen, as shown in Figure 3.

To increase effectiveness of EEG brain signal processing, filters have been developed to remove noise on brain signals, as shown in [75,76,77]. This pre-processing is outlined in Section 3.4. In addition, various data analysis techniques have been used to extract relevant information from EEG data such as cross-correlation and DL:

1. Cross-Correlation Function
  The most frequently used measure of interdependence between EEG signals in neuroscience is probably the cross-correlation function [78]. The cross-correlation function represents the inner product between two normalized signals and provides a measure of the linear synchronization or similarity between them [79]. Cross-correlation function combined with expert knowledge has been used, for example, in pattern recognition to correlate EEG frequency bands and other bodily signals, such as one’s heart rate for sleep classification [80], in neurophysiology to detect the risk level of schizophrenia [81] or to analyze the relationship of brain activity and breathing [82], and even as a calibration method for brain–computer interfaces [83].
2. Deep Learning
  Additionally, in the analysis of EEG signals, several approaches have been used that mostly consist of extracting features from the signals in several domains [84,85,86] that are selected by experts or by dimensional reduction algorithms, such as principal and independent component analysis [87] or more recently with differential entropy and linear discriminant analysis filters [88]. However, there is a fundamental inherent limitation in all these methods, as they require expert knowledge and manual expert manipulation of data is biased. Therefore, an automatic feature selection that is independent of human expertise is desirable.
  DL can serve this purpose, as is an artificial intelligence method that can learn features purely from data [89]. This method presents two main advantages: first, it learns features directly from the raw data using several layers (deep) in a hierarchical manner [90], and second, it can be applied to unlabeled data by unsupervised methods, this is without the need for expert supervision [91]. In general, DL architectures such as deep neural networks, contain an input layer and an output layer of ‘neurons’. In between, there are numerous layers of hidden units [92]. More specifically, deep neural networks use unsupervised learning to adjust the weights between hidden layers, enabling the network to identify the best internal features of the inputs [93].
  Recent research has involved DL techniques to classify EEG datasets of subjects’ executed movements [94,95,96] or motor imagery movements [97]. In addition, some contributions propose to use the EEG signals for DL biometric identification [98]. Also, there have been some results that are related to the identification of relevant sensors in emotion recognition EEG tests [99]. Recently scholars have used DL to perform human activity recognition from brain activity in Industry 4.0 environments [100] in which several transforms of raw data into images are depicted. Our research aims to expand this approach on the characterization of complex LM problem-solving behavioral patterns in an Industry 4.0 environment.

To achieve this, this study outlines the following four research hypotheses (H) and their related LM interpretation shown in Table 1. Furthermore, as these hypotheses are based on neurophysiological expert knowledge, management needs to be provided with tools that allow a proper discernment of which behavior is followed, based only on the data. For this reason, a DL-based soft sensor is developed that can perform this task.

## 3. Materials and Methods

To quantitatively test these hypotheses, as a first step to evaluate brain activity while exhibiting LM problem-solving behavioral patterns, such as *KATA* [11] and *(CPD)nA* [14], when dealing with a complex value streams, a case study is used.

As argued by [101], a single case study can be seen as only a possible building block in the process of developing validity and reliability of the proposed hypothesis. Following the recommendations of [102], a clear case-study roadmap is followed. This roadmap has several phases: (1) scope establishment (2) specification of population and sampling (3) data collection (4) standardization procedure and (5) data analysis.

### 3.1. Scope Establishment

**Figure 4:**
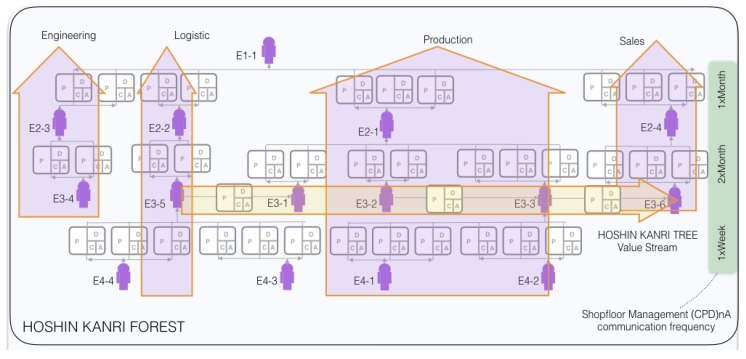
Part of the HOSHIN KANRI FOREST STRUCTURE [5].

EEG signals that Lean Managers generate within an organization when performing complex process LM optimization tasks are sought to be recorded. The organization selected for this case study is a Japan-based automobile manufacturing facility, embedded within a multinational corporation, where one of the authors has accompanied a systematic implementation of LM methodologies. The factory in which the study is carried out has 30 leaders in four hierarchical levels, and consists of 800 blue collars and 150 white collars. The LM matrix organizational design structure becomes evident when the continuous improvement shopfloor management HOSHIN KANRI FOREST reporting structure [4] is visualized in Figure 4: a PDCA-based LM network with a “vertical” hierarchy responsible for the allocation of resources (engineering, logistic, production, sales,...) that is balanced by a “horizontal” structures that connects the process owners along the value stream. In this manner, the organization is aligned to jointly achieve the corporate strategic objectives through continuous improvement. This ensures the systematic weekly training of organizational leaders in LM continuous improvement problem-solving routines.

The socio-cultural context in which the data collection is carried out is that of an experienced LM staff, with a corporate culture oriented towards continuous improvement throughout decades. The economic context of the automotive group in question follows a strategy of pressing cost reduction.

### 3.2. Specifications of Population and Sampling

Data were collected from 26 healthy male adult leaders (20–60 years of age with a mean of 40 years). None of them had a history of neurological or psychiatric disorder or was on chronic medication. All subjects were fluent in Japanese and had learned the language before they were age seven. Significant differences in EEG activity is usually found between right-handed and left-handed groups of subjects irrespective of the side of the brain considered [103]. Handedness was determined by the Edinburgh Handedness Inventory [104]. The initial group included 24 left-hemisphere-dominant persons (lateralization index of 29.5 ± 100%), one right-hemisphere-dominant person (−78.59%) and one ambidextrous person (+6.25%). Right-hemisphere-dominant and ambidextrous participants were excluded. The final sample included 24 male subjects with no significant differences in years of education, LM problem-solving experience, or handedness scores.

### 3.3. Data Collection

As previously shown, location and nomenclature of the 14 EEG electrodes is chosen as standardized by the American Electroencephalographic Society (AES) [74], are depicted in Figure 3 and marked in red [AF3, F7, F3, FC5, T7, P7, O1, O2, P8, T8, FC6, F4, F8, AF4].

**Figure 5:**
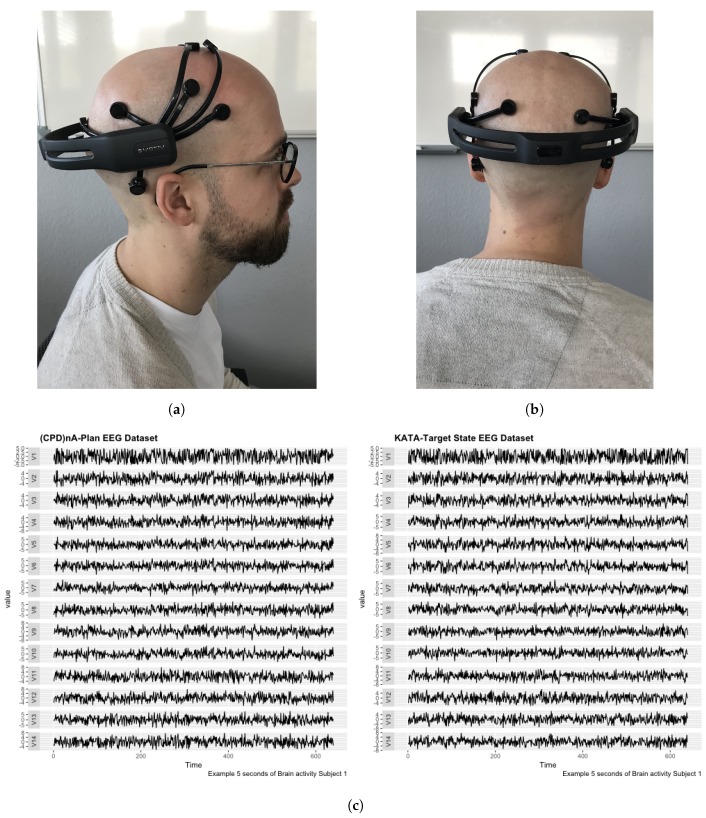
Data Collection. (**a**) EEG Low-Cost Portable Sensor. (**b**) EEG Low-Cost Portable Sensor. (**c**) 5 s recording time series multi-plot of first subject performing (CPD)nA-Plan (**a**) and KATA-III (**b**) of *Subject 1*.

The technical specifications of the EEG low-cost portable sensor shown in Figure 5c can be summarized as follows:

- Sampling method: Sequential sampling. Single ADC.
- Sampling rate: 128 samples per second (2048 Hz internal).
- Resolution: 14 bits 1 least significant beat = 0.51 μV (16-bit ADC, 2 bits instrumental noise floor discarded), or 16 bits.
- Bandwidth: 0.2–43 Hz, digital notch filters at 50 Hz.
- Filtering: Built in digital 5th order Sinc filter.
- Dynamic range (input referred): 8400 μV.
- Coupling mode: AC coupled.

To ensure best data collection and reduce hardware-related noise, the hair of all subjects was cut to <1 mm in length prior to measurement.

### 3.4. Data Pre-Processing

Initially, the raw data from the EEG is processed by applying a series of standard filters. Filtering such signals to remove artifacts is common in pre-processing these data, but may introduce temporary distortions in the signal [105]. The type of filter to choose depends essentially on the analysis of the dataset at hand. Filters can be causal, if they only include past and present information, while if they include past and future information, they are called non-causal. As in this case we are not interested in the timing of initial events it was decided to avoid non-causal filters at a price of introducing differences in the signal even before its onset at t = 0, due to backward filtering. An open access ***MATLAB*** toolbox for EEG, ***Fieldtrip***, was used [106]. ***Fieldtrip*** performs an infinite impulse response as default. An impulse response basically represents how the filter uses the unitary information of the signal in time. Infinite response filters produce an irregular shift at different frequencies, but they have a fundamental advantage in this case and that is that they are computationally very efficient. Summarizing, there are multiple criteria and trade-offs to take into account when designing and choosing digital filters [107]. The specific filters chosen were the following:

- First, a high-pass filter is first performed to remove the DC components from the signal (a cut-off of 1 Hz is considered sufficient and consistently produced good results in terms of SNR). [108]. This is because large drifts in the data were observed.
- Next, as indicated in the EEG sensor specifications, a hardware embedded low pass filter was implemented to eliminate frequencies above 50 Hz. This reduced the noise is associated with higher frequencies.
- Finally, in order to ensure the maximum level of anonymity for the subjects and to be scrupulous with the compliance standards of the company in which the study is carried out, a normalization in the range *[0.1]* of the values is performed. This can only be done because this study will not make comparisons between subjects.

### 3.5. Standardization Procedure

As exemplary shown in Figure 5c the subjects were placed individually in a room with 50 dBA artificially recreated large office noise and sat down to perform the tasks by writing down each step on an A3 sheet with paper and pencil. The tasks were completed without talking. The subjects sat in a reclining chair 20 cm away from the table so that the H-point, legs, and shoulders of the subject were fixed. This ensured that the position could be maintained in a defined replicable way and that only the arms, hands and the computer’s mouse were movable.

Each subject performed *KATA* and *(CPD)nA* behavioral tasks in a value stream that they owned and were therefore familiar with. The length of time allocated for the both *KATA* and *(CPD)nA* tasks was limited to 300 s each. The subject was instructed to not return to previous parts of the task. Specifically, within each task, there were pre-determined time intervals for each sub task. They were: in *KATA*, 10 s for Step 1 (KATA-I), 40 s for Step 2 (KATA-II), and 250 s for Step 3 and Step 4 (KATA-III), and in *(CPD)nA*, 10 seconds for Check, 250 s for Plan and 40 s for Do. This was established to ease the tagging of tasks for the subsequent analysis. The control task consists of writing the katakana syllabic alphabet for 30 s after finishing the problem-solving task. For purpose of example, a 5-s recording time series multi-plot of the first subject performing (CPD)nA-Plan (a) and KATA-III (b) is shown in Figure 5c.

Since no distinction can be made between subjects in terms of sex, LM experience, handedness score or training, and because the data collection procedure has been standardized as described, the data can be considered balanced. The complicacy of these datasets makes the need for a cross-correlation function and DL technique apparent if we are to analyze and inspect the dataset for relevant features.

### 3.6. Data Analysis

In this section, the soft sensors developed for the analysis is presented.

#### 3.6.1. Experimental Setup

The data analysis setup in this study were implemented with a computer equipped with an Intel(R) Xeon(R) Gold 6154 3.00 GHz CPU and an NVIDIA Quadro P4000 Graphic Process Unit (GPU) with 96 GB of random-access memory (RAM). The operating system was ***Red Hat Linux*** 16.04 64-bit version.

The training and testing of the DL model was carried out with ***Keras*** which is an interface to ***TensorFlow***. (Version 1.8), and the model was built in ***Python*** (Version 2.7) language [109]. TensorFlow is an interface for generating and executing machine learning algorithms, including training and inference algorithms for DNN models. Specifically, the TensorFlow TF.Learn module was adopted to create, configure, train, and evaluate the DNN. TF.Learn is a high-level Python module for distributed machine learning within TensorFlow and integrates a wide range of state-of-the-art machine learning algorithms. Additional Python interfaces were used: ***OpenCV*** for computer vision algorithms and image processing, ***Numpy*** for scientific computing and array calculation, and ***Matplotlib*** for displaying plots. The details of building the soft sensor model for problem-solving classification through EEG signals with Python are provided online at Open Access Repository and were created with ***Jupyter Notebook***.

#### 3.6.2. Deep Learning

**Figure 6:**
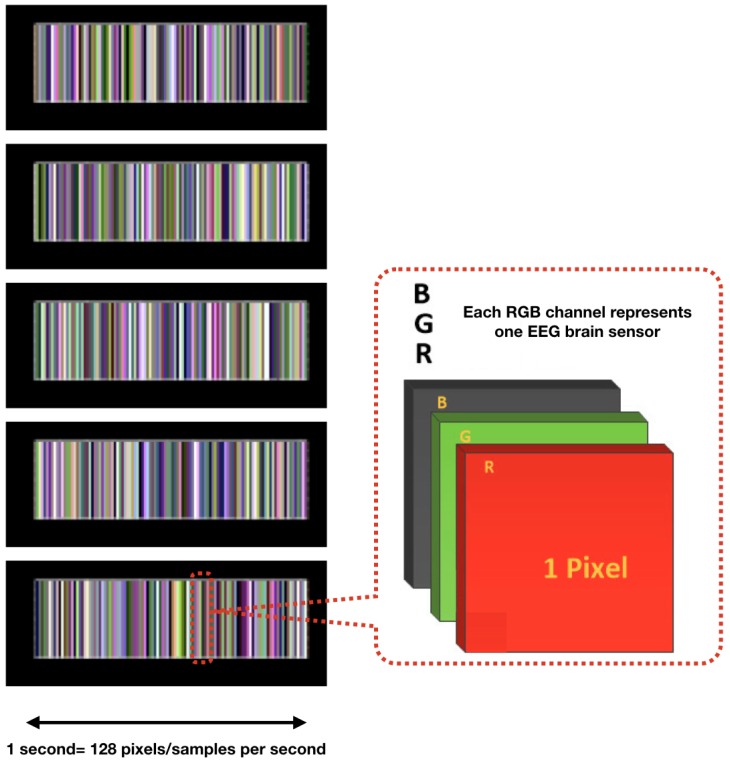
Multichannel Method [100].

**Figure 7:**
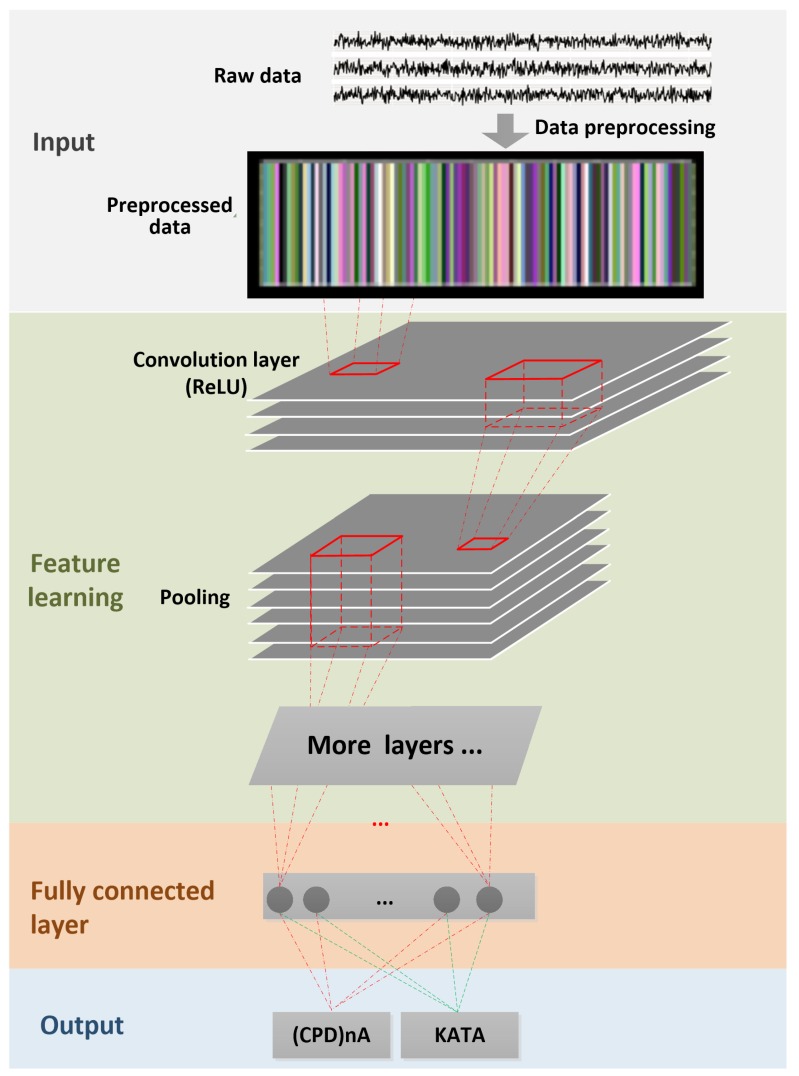
Deep-learning soft sensor for EEG classification.

1. Data Segmentation
  The time-dependent EEG data set is separated into 1-s segments during the data segmentation process. All subsequent operations, including feature extraction, classification, and validation, etc., are based on this previous segmentation. The nature of the segments depends on the application context and the sampling frequency of the EEG sensors. Increasing the length of the segments may improve the accuracy of the recognition, but the learning time will increase, and more time will be needed to obtain sufficient data. This could lead to delays in the response of applications in real time and restrict application scenarios [110].
2. Multichannel Method
  As described in [100], the multichannel data pre-processing method for DL treats data from three EEG channels as three superimposed color levels corresponding to red, green, and blue elements in the RGB color format. The EEG signal strength is projected to a corresponding color value in the [0.1] range. The three values of each point are represented as one pixel in the image. The resolution of the image is the same as the length of the segment (1 s/128 pixels because the sampling rate is 128 samples per second). The data collected from the different sensors are grouped in rows. The advantage of this method is that it greatly reduces the size of the image and results in a much shorter training time than that of the raw EEG time series analysis, and does not require expert knowledge. Figure 6 shows the principle of the application of this method and an example image. The data fragment used in this figure shows the first five seconds of the one used in Figure 5c.
3. Deep-Learning Soft Sensor Architecture
  After pre-processing and segmentation, the original data segments are transformed into images, to which the DL methods are applied. In this study, the deep convolutional neural network algorithm is used [111]. This model has its own parameters, such as the number of convolutional layers, the learning rate, pooling size, etc. Figure 7 shows the DL soft sensor architecture and workflow. The first layer to extract features from an input image is the convolution layer. It preserves the relationship between pixels by learning image features using small squares of input data. The Rectified Linear Unit (ReLU) method is used for the non-linear operation to introduce non-linearity in the DL model. Following the convolution layer is the pooling layer which can reduce the dimensionality size. The max pooling method is used in our model, which takes the largest element from the rectified feature map. Multiple convolution layers and pooling layer can be added to the DL model to obtain the best performance. Finally, the feature map matrix produced by the convolution and pooling layers is flattened and fed into the fully connected layer to output the classes using the SoftMax activation function. Following the approach in [110], in order to classify between the LM behavioral patterns, the shallow features are merged with the deep-learned features on the last fully connected layer, as shown in Figure 7. More details of the DL models are available online at Open Access Repository.

## 4. Results and Discussion

In this section, the experimental results of the analysis are presented, their interpretation as well as the experimental conclusions that can be drawn from them. To do so, one by one on all the hypotheses presented in Table 1 are checked and commented by using the cross-correlation function, and the results of the automatic characterization of LM problem-solving behavioral patterns by means of the DL soft sensor are presented:

### 4.1. Results and Discussion of Cross-Correlation Function

**Figure 8:**
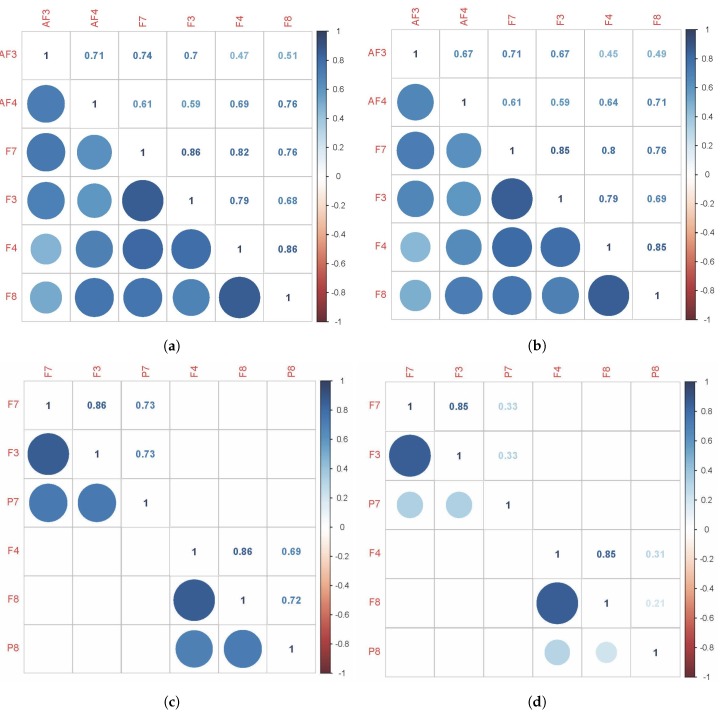
Graphical representation of Hypotheses confirmation with *Subject 1* results. (**a**) (CPD)nA Results. Sensors *AF3-F7-F3-AF4-F4-F8*. (**b**) KATA Results. Sensors *AF3-F7-F3-AF4-F4-F8*. (**c**) (CPD)nA Results. Sensors *F7-F3-P7-F4-F8-P8*. (**d**) KATA Results. Sensors *F7-F3-P7-F4-F8-P8*.

1. Corresponding to *H1*
  All sensors *AF3-F7-F3-AF4-F4-F8* are expected to correlate with each other, although the strength of correlations may differ, which means that the task is *executive*.
  Figure 8a,b shows the cross-correlation among sensors *AF3-F7-F3-AF4-F4-F8* of *Subject 1*. These results show that for the same subject, all sensors *AF3-F7-F3-AF4-F4-F8* presented a correlation that exceeds 0.45. This supports *H1* which stated that subjects engaging in LM problem-solving behavioral patterns present a strong correlation in their PFC activity.
2. Corresponding to *H2*
  Sensors *F7* and *F3*, as well as sensors *F4* and *F8*, are expected to present stronger correlation, which means that the task is *goal driven*.
  Figure 8c,d shows that the correlations between sensors *F7* and *F3*, as well as sensors *F4* and *F8*, are stronger than others, which exceed 0.85. This supports *H2* which stated that subjects engaging in LM problem-solving behavioral patterns present a coordinated a dorsolateral- and ventromedial PFC activity.
3. Corresponding to *H3*
  Sensors *F7* and *P7*, as well as sensors *F8* and *P8*, are expected to present no correlation or a very weak correlation.
  The results show that for *KATA*, the correlations between sensor *P7* to sensors *F7* and *F3* (0.21–0.33), and sensor *P8* to sensors *F4* and *F8* between 0.21 and 0.34, are much weaker than *(CPD)nA*. This supports *H3* which stated that goal-oriented, context-independent LM problem-solving behavioral patterns would present a low correlation between the dorsolateral PFC and the TPJ.
4. Corresponding to *H4*
  Sensors *F7* and *P7*, as well as sensors *F8* and *P8*, are expected to present a strong correlation.
  The result shows that for *(CPD)nA*, sensor *P7* is correlated with sensors *F7* and *F3* between 0.69 and 0.73, sensor *P8* is correlated with sensors *F4* and *F8* between 0.68 and 0.73. This supports *H4* which stated that goal-directed, context-dependent LM problem-solving behavioral patterns would present a high correlation between the dorsolateral PFC and the TPJ.

**Figure 9:**
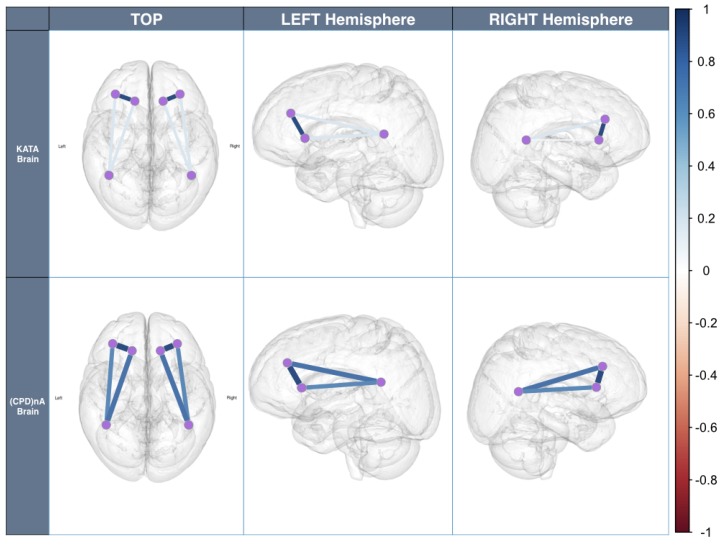
Brain Overlay of cross-correlation among sensors *F7, F3* and *P7*, as well as *F4, F8* and *P8* of Subject 1.

Hypotheses *H1*, *H2*, *H3* and *H4* were verified by calculating the cross-correlation among sensors for both *KATA* and *(CPD)nA* LM behavioral patterns:

For clarity, Figure 9 depicts Figure 3b results on a brain layout. This shows how while performing both LM problem-solving behavioral patterns, the PFC shows a highly coordinated activity. When *KATA*, a goal-directed context-independent behavioral pattern is performed, the coordination between the dorso-lateral prefrontal cortex and the TPJ is non-existent. This changes when *(CPD)nA*, a goal-directed context-dependent behavioral pattern is performed.

***In summary, all hypotheses were tested and verified***. Subjects under scrutiny presented a high PFC activity and a high correlated dorsolateral PFC and vm-PFC activation. The combination of these factors enabled us to label such LM problem-solving behavioral patterns as executive and goal-oriented. Furthermore, *KATA* did not present a dl-PFC and TPJ modulation, whereas *(CPD)nA* did. This allows us to label *KATA* as context-independent and *(CPD)nA* as context-dependent behavioral pattern. These results were validated by a DL predictor algorithm at very high levels of accuracy.

### 4.2. Results and Discussion of Deep-Learning Soft Sensor

After testing and verifying the hypotheses, the results of a ***DL soft sensor that can characterize the LM problem-solving behavioral task with a 99% of accuracy*** are presented. This is important, because it is not necessary to have an expert knowledge of neurophysiology to discern whether a certain LM problem-solving behavioral pattern is of one nature or another. The very nature of the DL soft sensor will determine it automatically.

As shown in the Open Access Repository, using Keras, TensorFlow backend for the DNN and OpenCV/Numpy for the image manipulation, a dataset of ***12,000 images*** is used. As a standard procedure, the data is split into ***training dataset*** of 20 Subjects (80%), ***testing dataset*** of 2 Subjects (10%) and ***validation dataset*** of 2 Subjects (10%). These subjects are chosen randomly between the sample of 24 Subjects.

**Figure 10:**
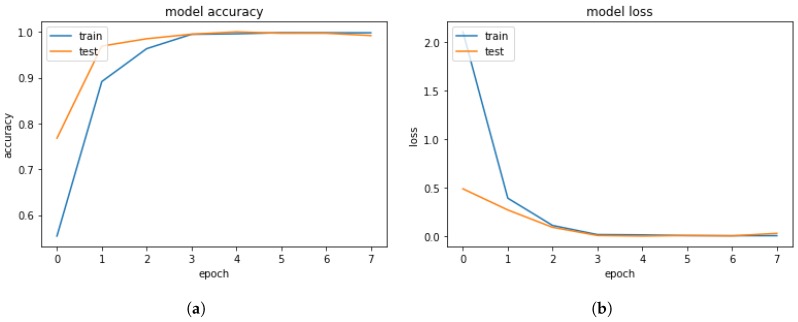
DL Training and Testing Results. (**a**) DL Model Training Accuracy. (**b**) DL Model Training Loss.

The ***training dataset*** is used to train the DNN throughout several epochs as shown in Figure 10. It can be observed that both accuracy and loss do not increase or decrease significantly after epoch number 4.

The ***testing dataset*** is subsequently used to test DNN performance. The confusion matrix is a standard procedure to summarize the results of such a training by typically combining contingency classes (TRUE, FALSE) and (OK, not-OK), hence building four categories:

1. True Negative *(TN)*, which is an error and has been predicted as an error
2. False Positive *(FP)*, which is an error but has not been predicted as an error, and is by far the most damaging category
3. False Negative *(FN)* which is not an error but has been predicted as an error
4. True Positive *(TP)* which is not an error and has not been predicted as an error.

**Figure 11:**
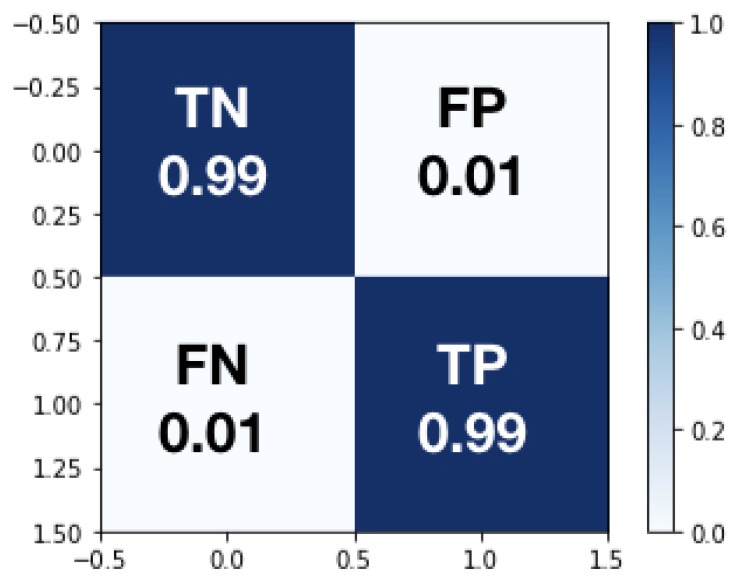
DL Model Testing Confusion Matrix.

The results are summarized in Figure 11. Specifically, given the balanced dataset chosen, the *accuracy* (ACC) delivered by the DNN soft sensor, defined by the expression ACC=(TP+TN)/(TP+TN+FP+FN), is 99%. The *TN* rate is 99%, the *TP* rate is 99%, the *FN* rate is 1% and the *FP* rate is also 1%. These levels of *ACC* can be considered acceptable for such a complicated industrial classification problem.

## 5. Management Conclusions and Future Steps

These results allow for different ways of further industrial implementation. To do so, these results must be interpreted in a broad context of Industry 4.0. This section provides some essential aspects that will help to understand and contextualize the contributed results through a meta-discussion at various organizational levels.

To attain operational excellence, leaders need to better understand their people. At the verge of empirical psychological neuroscience, organizational behavioral theory, and artificial intelligence, this multidisciplinary paper seeks to help organizational leaders, LM practitioners and scholars to develop a better understanding of the brain’s dynamics that are associated with certain standard LM problem-solving behavioral patterns that are commonly found in corporate settings.

The empirical results have provided evidence to assume that from a neurological perspective, it is possible to provide organizational leaders with certain conclusions and take-offs for future endeavors:

- The LM tasks studied can be regarded as goal-oriented tasks due to the highly coordinated activity of the dorsolateral and ventromedial PFC. This means that organizational leaders who exhibit such problem-solving behavioral patterns are intending to attain certain goals and perform a cerebral internal modulation of those goals. The immediate consequence is that strategic goals such as operational excellence are more likely to be achieved when implementing LM.
- LM tasks can be regarded as executive tasks that are guided mainly by the PFC. This means that organizational leaders, when dealing with such problem-solving behavioral patterns, consistently exercise decision-making, working memory, and self-control while performing LM. The consequence is that LM is provably a managerial conglomerate that induces and executive cerebral state and therefore, organizational leaders that decide to implement LM within their organizations and setting them in a systematic path of execution towards operational excellence.
- The LM problem-solving behavioral pattern, *KATA*, after definition of the target states apparently induces the subjects into a mental state in which information that is not relevant to the target-state achievement is not taken into consideration. This is shown by the lack of coordinated activity between PFC areas and the TPJ. This has powerful implications for the operations management community. It could mean that target-state setting would induce subjects into undesirable inflexible problem-solving behavioral patterns in which the decision-making process is not modulated by the complexity of ever-changing organizational value-stream settings. Individuals could make decisions independently of their context to serve their individual targets. This could potentially not serve a higher organizational alignment. In highly complex organizational settings where interdependent behavior is essential for organizational alignment, this could have dire consequences.
- In contrast, the LM problem-solving behavioral pattern (CPD)nA-Plan, which advocates only continuous improvement without target conditions, seems to enable cerebral modulation of the PFC activity by providing for coordination with the TPJ. In highly complex environments where interdependent value-stream constraints are to be simultaneously considered, such an LM behavioral trait seems to permit the flexibility that is necessary for a coordinated organizational effort towards the demands of alignment and, from a cerebral perspective, offers a better promise to ensure individual and organizational fitness.

In an Industry 4.0 context, EEG sensor signals placed on human process owners combined with DL soft sensor architectures within Industry 4.0 environments could have an impact at various levels of aggregation in value chains.

1. EEG combined with DL at a shoopfloor level shall impact quality, reliability, and cost.
  In an Industry 4.0 shopfloor environment, in which man and machine interact constantly to create value, it is essential that they communicate effectively and efficiently in real time. The creation of intelligent algorithms capable of characterizing the complex behaviors of the human brain and making them understandable to the machine seems of vital importance to ensure a symbiosis that increases machine efficiency and human effectiveness.
  Future lines of research should try to better understand how the human brain can integrate its work into the Industry 4.0 shopfloor by means of brain sensors, making possible the cerebral interface between man and machine without the need for low-bandwidth elements such as touch screens or verbal commands.
  The DL-based algorithms based on process EEG signals presented in this paper can be a spearhead that allows the classification and training of Industry 4.0 intelligence systems that allow this integration. This intelligence integrated in the value streams will allow humans and machines to co-exist in a way in which artificial and human intelligence will complement each other, thus increasing the process capability of generating higher standards of quality, reliability, and ultimately reducing cost.
2. EEG combined with DL at a strategic manufacturing system level.
  The DL characterization of LM problem-solving behavioral patterns is expected to help Industry 4.0 leaders in their choice of adequate manufacturing systems and their related problem-solving methods in their future pursuit of strategic organizational goals.
  As demonstrated by the presented DL algorithms, no neurophysiological expert knowledge is necessary to discriminate between two different complex LM problem-solving behavioral patterns performed by Industry 4.0 process owners. This could help future industry leaders make better decisions about which manufacturing systems to choose from a neurological point of view. This bottom-up approach is novel in the field of management and represents in itself a breakthrough in the study of manufacturing systems in Industry 4.0 environments.
  DL-based applications combined with multiple simultaneous EEG measurements to different actors during the performance of different complex tasks such as decision-making, data analysis, leadership interactions with subordinates, or other relevant actors, could lead to new revelations in the field of *neuroeconomics* among other fields. Likewise, by establishing a feedback loop to the leadership process of each individual, this knowledge could provide specific knowledge of each individual during their interaction with other stakeholders. This could mean a breakthrough towards a customization of leadership and towards a transformation of business culture from the neuroscientific knowledge of human behavior in an Industry 4.0 environment.

As a final note, a word of caution. Although the results are promising, no premature conclusions of causality should be drawn in any way. For several reasons: first, blinking and eye movement produce strong electrical impulses that can affect EEG measurement. Therefore, in future research, as subjects perform tasks with their eyes open in Industry 4.0 environments, pre-processing using an electro-oculogram as an adaptive noise canceller may be necessary. Second, subjects were asked to cut their hair to <1 mm in length prior to measurement to facilitate signal recording and consequently increase algorithm’s performance. This is a rather unrealistic condition for an Industry 4.0 setting. Third, group analysis would have been desirable. However, in this study focus on inter-subject correlations was not possible mainly for one reason: compliance rules of the organization in which the study was carried out, did not allow the researchers to compare the results from different subjects in order to avoid labor-related conflicts. For this reason, only one subject was exemplary displayed in Figure 8 and Figure 9. Fourth, the population used for the study was relatively small, quite homogeneous, focused on one technological set of problems and drawn from only one geographical region. Furthermore, the level of LM expertise, age, or gender could be aspects to be controlled for and/or used as a covariate or explanatory variable in future research.

## Figures and Tables

**Table 1:** Research questions and related hypotheses.

#	Hypotheses	LM Interpretation
1	*H1*. Subjects that engage in LM problem-solving behavioral patterns present a strong correlation in their PFC activity. In such a case, datasets from sensors *AF3-F7-F3-AF4-F4-F8* would present a strong correlation.	This would mean that LM problem-solving behavioral patterns can be understood in neurological terms as executive behavioral pattern.
2	*H2*. Subjects that engage in *KATA* and *(CPD)nA* present strong correlations of their dorsolateral PFC-ventromedial PFC combined activity. In such a case, datasets from sensors *F7* (ventromedial PFC Left Hemisphere) and *F3* (dorsolateral PFC Left Hemisphere), as well as *F4* (ventromedial PFC Right Hemisphere) and *F8* (dorsolateral PFC Right Hemisphere), would present a strong correlation.	This would mean that both *(CPD)nA* and *KATA* can be regarded in neurological terms as goal-oriented LM behavioral pattern.
3	*H3*. Subjects that engage in *KATA* present weakly dorsolateral PFC-TPJ correlated combined activity. In such a case, datasets from sensors *F7* (ventromedial PFC Left Hemisphere) and *F3* (dorsolateral PFC Left Hemisphere) would not correlate strongly with *P7* (TPJ Left Hemisphere), and sensors *F4* (ventromedial PFC Right Hemisphere) and *F8* (dorsolateral PFC Right Hemisphere) would not correlate strongly with *P8* (TPJ Right Hemisphere).	This would mean that *KATA* could be understood in neurological terms as a goal-oriented, context-independent LM behavioral pattern.
4	*H4*. Subjects that engage in *(CPD)nA* present a strong dorsolateral PFC-TPJ correlated combined activity. In such a case, datasets from sensors *F7* (ventromedial PFC Left Hemisphere) and *F3* (dorsolateral PFC Left Hemisphere) would correlate strongly with *P7* (TPJ Left Hemisphere), and sensors *F4* (ventromedial PFC Right Hemisphere) and *F8* (dorsolateral PFC Right Hemisphere) would correlate strongly with AF3 (dorsolateral PFC Left Hemisphere).	This would mean that *(CPD)nA* could be understood in neurological terms as a goal-oriented, context-dependent LM behavioral pattern.

## Abbreviations

The following abbreviations are used in this manuscript:

LM	Lean Management
SNR	Signal-to-Noise Ratio
PFC	Prefrontal Cortex
PDCA	Plan-Do-Check-Act
(CPD)nA	Check-Plan-Do-...-Act
EEG	Electroencephalography
DL	Deep Learning
H	Hypothesis
